# Barriers and facilitating factors to the uptake of antiretroviral drugs for prevention of mother-to-child transmission of HIV in sub-Saharan Africa: a systematic review

**DOI:** 10.7448/IAS.16.1.18588

**Published:** 2013-07-19

**Authors:** Annabelle Gourlay, Isolde Birdthistle, Gitau Mburu, Kate Iorpenda, Alison Wringe

**Affiliations:** 1 Department of Population Health, Faculty of Epidemiology and Population Health, London School of Hygiene & Tropical Medicine, London, United Kingdom; 2 International HIV AIDS Alliance, Hove, United Kingdom; 3 Division of Health Research, Lancaster University, Bailrigg, United Kingdom

**Keywords:** HIV, vertical transmission, prevention, barriers, review, Africa

## Abstract

**Objectives:**

To investigate and synthesize reasons for low access, initiation and adherence to antiretroviral drugs by mothers and exposed babies for prevention of mother-to-child transmission (PMTCT) of HIV in sub-Saharan Africa.

**Methods:**

A systematic literature review was conducted. Four databases were searched (Medline, Embase, Global Health and Web of Science) for studies conducted in sub-Saharan Africa from January 2000 to September 2012. Quantitative and qualitative studies were included that met pre-defined criteria. Antiretroviral (ARV) prophylaxis (maternal/infant) and combination antiretroviral therapy (ART) usage/registration at HIV care and treatment during pregnancy were included as outcomes.

**Results:**

Of 574 references identified, 40 met the inclusion criteria. Four references were added after searching reference lists of included articles. Twenty studies were quantitative, 16 were qualitative and eight were mixed methods. Forty-one studies were conducted in Southern and East Africa, two in West Africa, none in Central Africa and one was multi-regional. The majority (*n*=25) were conducted before combination ART for PMTCT was emphasized in 2006. At the individual-level, poor knowledge of HIV/ART/vertical transmission, lower maternal educational level and psychological issues following HIV diagnosis were the key barriers identified. Stigma and fear of status disclosure to partners, family or community members (community-level factors) were the most frequently cited barriers overall and across time. The extent of partner/community support was another major factor impeding or facilitating the uptake of PMTCT ARVs, while cultural traditions including preferences for traditional healers and birth attendants were also common. Key health-systems issues included poor staff-client interactions, staff shortages, service accessibility and non-facility deliveries.

**Conclusions:**

Long-standing health-systems issues (such as staffing and service accessibility) and community-level factors (particularly stigma, fear of disclosure and lack of partner support) have not changed over time and continue to plague PMTCT programmes more than 10 years after their introduction. The potential of PMTCT programmes to virtually eliminate vertical transmission of HIV will remain elusive unless these barriers are tackled. The prominence of community-level factors in this review points to the importance of community-driven approaches to improve uptake of PMTCT interventions, although packages of solutions addressing barriers at different levels will be important.

## Introduction

In 2008, 12 million women aged 15 years and over were estimated to be living with HIV in sub-Saharan Africa, and of the 330,000 new HIV infections among children (under 15) globally in 2011, over 90% were in sub-Saharan Africa [1, 2]. The vast majority of new HIV infections among children occur through mother-to-child transmission (MTCT).

Antiretroviral therapy (ART) is the core intervention of the prevention of mother-to-child transmission (PMTCT) service package (programme “prong 3,” concerning interventions to reduce vertical transmission among HIV-positive pregnant women, alongside other “prongs” covering HIV prevention in women of reproductive age, family planning, and long-term HIV care and treatment [3]). Antiretroviral (ARV) drugs can reduce the likelihood of HIV vertical transmission from 15 to 45% in the absence of any intervention, to <5% [3]. In 2010, the World Health Organisation (WHO) published guidelines advising that all HIV-positive pregnant women with CD4 counts <350 cells/mm^3^ should initiate combination ART for their own health (herein referred to as “cART”) [1], although some countries in sub-Saharan Africa still use lower CD4 count thresholds [4]. For those with higher CD4 counts, antiretroviral prophylaxis in pregnancy is advised, with variations in drug regimens and duration depending on the option (A, B or B +) adopted in each country (Table 1) [5, 6]. PMTCT treatment guidelines have evolved considerably over time in sub-Saharan Africa, following the first recommendation for ARV drugs for PMTCT in 2000 (short-course prophylaxis starting late in pregnancy or during labour, including single-dose nevirapine (NVP) for mothers and infants) [7], and subsequent revisions in 2004 (standardization and simplification of regimens) and 2006 which emphasized the importance of providing cART to pregnant women for their own health (cART for those with CD4 counts below 200 cells/mm^3^, or azidothymidine (AZT) prophylaxis starting from 28 weeks of pregnancy, single-dose NVP during labour and delivery, and infant prophylaxis for one week) [8, 9]. The 2010 recommendations include an option (B) that unifies prophylaxis for PMTCT of HIV and treatment for an HIV-positive woman's own health. Option B+ (ARV therapy for all HIV-positive pregnant women continued for life) is expected to be formally recommended in 2013, with foreseen benefits including further simplification and operational simplicity, avoidance of stopping and starting ARV drugs, protection against vertical transmission in future pregnancies and protection against sexual transmission to sero-discordant partners [5].

**Table 1:** ARV treatment guidelines for prevention of mother-to-child transmission of HIV

	Option A	Option B	Option B+
Mother (CD4≤350 cells/mm^3^)	Triple ARVs, starting from diagnosis and continued for life	Triple ARVs, starting from diagnosis and continued for life	Triple ARVs regardless of CD4 count, starting from diagnosis and continued for life
Mother (CD4>350 cells/mm^3^)	Prophylaxis: *Antepartum:* AZT from 14 weeks gestation *Intrapartum:* sd NVP at onset of labour and AZT/3TC *Postpartum:* AZT/3TC for seven days	Prophylaxis: Triple ARVs from 14 weeks gestation until one week after exposure to breastmilk has ended	
Infant	NVP (daily) from birth until one week after cessation of breastfeeding, or until age four to six weeks if replacement feeding	NVP or AZT (daily) from birth until age four to six weeks (regardless of infant feeding method)	NVP or AZT (daily) from birth until age four to six weeks (regardless of infant feeding method)

Adapted from ref. [5]. ARV=antiretroviral; AZT=azidothymidine; NVP=nevirapine; sd=single-dose.

Although coverage of ARVs for PMTCT has increased, and some high-income regions have nearly achieved universal coverage, only 53% of pregnant women and 35% of infants in sub-Saharan Africa in need of ARVs for PMTCT received the treatment in 2009 (increased from 15 and 12% in 2005, respectively) [1].

While there is a greater understanding of barriers to ARV usage in the context of HIV treatment programmes in general [10, 11], less is known about the issues faced specifically by pregnant women. Reasons for low uptake (herein used to include access, initiation and adherence) of ARVs for PMTCT are emerging, but the results have not been comprehensively synthesized.

Two reviews, conducted between 2011 and 2012, focussed primarily on the magnitude of uptake and levels of adherence to ARVs during and after pregnancy, rather than associated factors, and excluded qualitative studies which may offer important insights regarding reasons for poor uptake [12, 13]. Other related reviews, conducted between 2009 and 2011, focussed more specifically on linkage and retention of HIV-positive pregnant women in care and treatment services [14], community-based interventions for PMTCT [15], and summarized achievements and failures of PMTCT services in specific regions (West Africa) [16]. To our knowledge, no studies have systematically reviewed barriers to PMTCT ARV uptake (both prophylaxis and cART) in sub-Saharan Africa, from both a qualitative and quantitative perspective.

**Figure 1:**
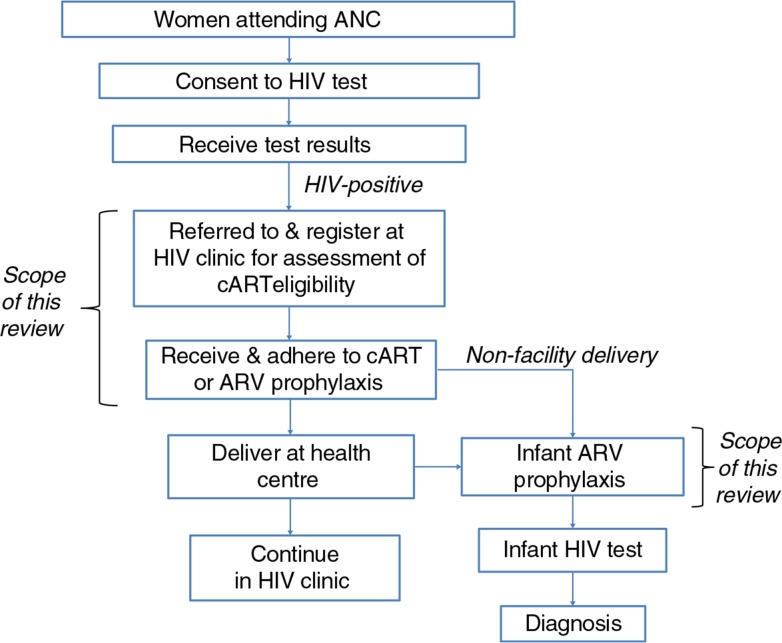
Scope of this review in relation to the PMTCT continuum of care for HIV-positive women and their infants. The narrowing of boxes reflects the attrition in terms of numbers of women and infants through the steps. In different service delivery models, cART or ARV prophylaxis may be received either at the ANC or HIV clinic. ANC=antenatal clinic; ARV=antiretroviral; cART=combination antiretroviral therapy for own health.

There is an urgent need to understand the reasons for low uptake of this intervention in order to prioritize strategies to enhance PMTCT programme uptake, and accelerate progress towards the United Nations Global Plan targets (eliminate new HIV infections in children and sustain lives of mothers) [17] and Millennium Development Goals 4 (child health), 5 (maternal health) and 6 (HIV/AIDS) [18]. A systematic literature review was therefore conducted to inform HIV programming, by drawing together existing information on barriers and facilitating factors to the uptake of ARVs for PMTCT in sub-Saharan Africa (Figure 1 illustrates the focus of this review within the PMTCT continuum of care).

## Methods

### Search strategy

Four databases were searched (Medline, Embase, Global Health and Web of Science), combining terms related to HIV, PMTCT, ARVs and barriers/uptake (supplement 1a–d). The search was limited to studies conducted in sub-Saharan Africa and published in English from January 2000 (when PMTCT/antiretroviral treatment programmes were introduced in this region) to September 2012. Retrieved references were imported into EndNote X5 then duplicates were removed. Reference lists of included articles were searched.

### Study selection

Titles and abstracts were screened by one researcher (AG) using pre-defined criteria (Table 2). Both qualitative and quantitative studies were eligible, as well as mixed-methods designs. Ten percent of titles and abstracts (randomly selected) were screened by a second researcher (AW) to verify inclusion decisions. Uncertainties were resolved through discussion between both reviewers. Authors of five studies were contacted to clarify methods and results.

**Table 2:** Inclusion and exclusion criteria for quantitative, qualitative and mixed-methods studies

**All study designs**	
Excluded	Location: Not conducted in sub-Saharan Africa
	Publication type: Reviews, commentaries and editorials
**Quantitative studies**	
Included	Analysis of factors associated with any of the following outcomes: maternal and/or infant receipt or use of ARV prophylaxis maternal cART initiation (or adherence), or maternal registration at the ART clinic, during pregnancy Participants: HIV-positive women (pregnant or with previous experience of the PMTCT programme) and their infants
Excluded	cART initiation among HIV-positive children (outcome) Referral to HIV care and treatment after exit from the PMTCT programme (outcome) Uptake of ARVs for PMTCT over time (time period as the explanatory variable) Studies that did not report a multivariate analysis (did not adequately control for confounding), or gave insufficient information on statistical methods to reach a decision
**Qualitative studies**	
Included	Specifically explores barriers or facilitating factors related to any of the following: receipt or use of maternal or infant ARV prophylaxis cART during pregnancy , or referral to HIV care and treatment during pregnancy challenges to delivering the components (above) of the PMTCT programme Participants: Any with experience or perceptions of the PMTCT programme
**Mixed methods studies**	
Included	Either qualitative or quantitative component meets inclusion criteria above

Hierarchy applied to exclusions: (1) Location; (2) included outcomes not reported, or publication type; (3) included outcomes reported but no associated factors, or excluded factor (time period); (4) included outcomes/explanatory variables but no multivariate analysis/brief methods. ARV=antiretroviral; cART=combination antiretroviral treatment; PMTCT=Prevention of mother-to-child transmission.

### Quality appraisal

Quantitative studies were excluded if they did not control for confounding through multivariable analysis. Those meeting inclusion criteria were further appraised for quality and a sensitivity analysis was conducted.

Qualitative studies were not excluded on the basis of quality as there are no objective methods or evidence base for guiding such decisions, so a quality appraisal and sensitivity analysis was performed, using the approach of Thomas and Harden [19]. A qualitative appraisal checklist and scoring scheme was adapted from the Critical Appraisal Skills Programme (CASP) tool [20] by two reviewers (AG and IB), after reviewing relevant tools and literature [10, 19–24]. The adaptation aimed to: cover the core evaluative criteria/main quality issues identified by Cohen *et al*. and Thomas and Harden [19, 21], and include objective and specific questions that could be answered “yes” or “no” (supplement 2a). One point was awarded for each “yes,” for a total of 16 points. A quantitative appraisal checklist, based on CASP and “Strengthening Reporting of Observational studies in Epidemiology” (STROBE) tools and using a similar (17 point) scoring system, was also developed (by AG, IB, AW) to cover multiple quantitative designs (supplement 2b) [20, 25].

One researcher (AG) applied the checklists to all the included qualitative or quantitative literature (including applicable components of mixed-methods designs). For quality control, 25% of the qualitative studies and 30% of the quantitative studies were double-marked (by AW and IB). There was agreement on 5/6 double-marked qualitative studies and 6/7 quantitative studies, in terms of their inclusion/exclusion during sensitivity analyses; after discussion the qualitative study was retained while the quantitative study was excluded.

Studies (quantitative or qualitative) with scores below 10 were considered *a priori* to be of lower quality. In the sensitivity analyses, results and conclusions were re-considered after removing findings of lower quality studies.

### Data extraction and analysis

Data were extracted from qualitative studies using thematic synthesis [19]. Quotations and descriptions of findings in results sections and abstracts were potential “data.” Factors were only recorded if they were specifically related to PMTCT ARV uptake: barriers to other PMTCT cascade steps were not recorded. Codes were then categorized to build descriptive themes. Quantitative results were categorized using the same broad headings. Any factors analyzed with regards to PMTCT ARV use (as defined in Table 2) were listed, and the results of statistical tests for association were noted. Data were only extracted from components of mixed-methods studies that satisfied inclusion criteria. Findings from data collected prior to and after 2007 were compared to gauge whether barriers and facilitating factors changed over time, following updates in WHO recommendations in 2006.

## Results

### Characteristics of included studies

**Figure 2:**
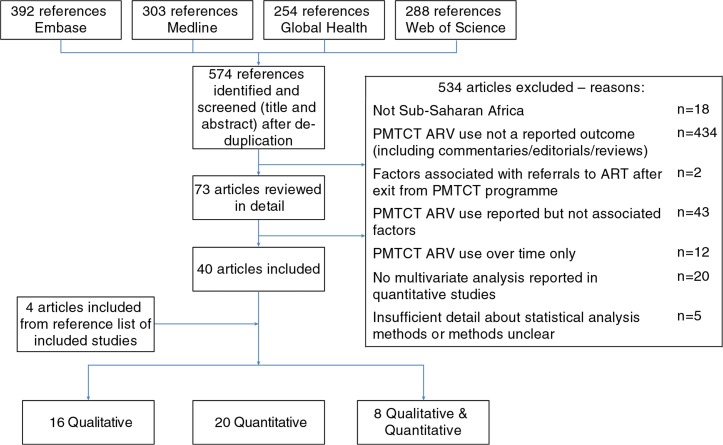
Flow diagram of systematic search results.

A total of 574 references were identified, of which 44 articles met the inclusion criteria. Four were added after searching reference lists (Figure 2). There was agreement on 56/58 of the double-screened references. Twenty studies were quantitative (primarily cross-sectional or cohort), 16 were qualitative and eight used mixed methods. Overall, the studies were conducted in 12 different countries, with most in Southern (*n*=13) and East Africa (*n*=28), few in West Africa (*n*=2), none in Central Africa and one mixed-region study. More studies were conducted in urban (*n*=21) than rural (*n*=10) settings (11 in both rural and urban settings, two settings unclear). The majority of studies were conducted during early phases of PMTCT programmes, before the introduction of cART for PMTCT, although an increasing amount of literature has emerged recently (approximately 1/3 included studies were published since January 2011). Tables [3–5] summarize the included studies.

**Table 3:** Characteristics of qualitative studies included

#	Author, year	Setting	Study design	Participants	Sample size
1	Awiti, 2011	Kenya, Urban and rural	Narratives	HIV+ pregnant women on cART	28; 16 rural and 12 urban
2	Burke, 2004	Tanzania, Urban and rural	IDIs and FGDs	Health workers, pregnant women, HIV+ individuals, other men and women	12 interviews; 5 FGDs
3	Chinkonde, 2009	Malawi, Urban and rural	IDIs and FGDs	HIV+ women (sub-sample of cohort at PMTCT sites); husbands	28 IDIs; 4 FGDs of 6–9 per group (28 total); 12 IDIs with men
4	Delva, 2006	South Africa, Urban	IDIs	Key informants	14
5	Duff, 2010	Uganda, Urban and rural	IDIs and FGD	HIV+ mothers (registered in PMTCT programme)	45 interviews, 1 FGD (8 women)
6	Duff, 2012	Uganda, Urban and rural	FGDs	Men (married/with female partners)	40 participants in 4 groups
7	Kasenga, 2010	Malawi, Rural	IDIs	HIV+ women (registered in PMTCT programme)	24
8	Levy, 2009	Malawi, Urban	IDIs, FGDs, observations	HIV+ women (participating in PMTCT programme), key informants	IDIs: 34 women, 21 key informants; FGDs: 21 women (4–6 per group)
9	Nkonki, 2007	South Africa, Urban and rural	IDIs	HIV+ women (sub-sample of cohort study on PMTCT)	58
10	O'Gorman, 2010	Malawi, Rural	IDIs and FGDs	Ante/post-natal women, fathers, grandmothers, TBAs, health workers, community leaders	44 in FGDs in total, 26 interviews
11	Painter, 2004	Cote d'Ivoire, Urban	IDIs	HIV+ women (discontinued/refused PMTCT follow-up visits)	27
12	Sprague, 2011	South Africa, Urban	IDIs (and patient file review)	HIV+ women, female carers of HIV+ children, key informants	83 women, 32 carers, 38 key informants.
13	Stinson, 2012	South Africa, Urban	IDIs (structured)	Pregnant/post-natal HIV+ women (on cART or eligible for cART)	28 women; 21 health workers
14	Theilgaard, 2011	Tanzania, Urban	IDIs, FGDs, and observations	HIV+ women; health care providers	FGDs: 12 women; 6 HWs. IDIs: 18 women
15	Towle, 2008	Lesotho, Urban and rural	IDIs and participant observation	Health workers; HIV programme staff; women/men (reproductive age); grandmothers	29 (total)
16	Winestone, 2012	Kenya, Rural	IDIs	Health care providers	36

# Study number (sequential order; differs from bibliographic reference number); IDI=In-depth interview; FGD=Focus group discussion; NVP=nevirapine; TBA=traditional birth attendant; ANC=antenatal clinic; cART=combination antiretroviral therapy; PMTCT=Prevention of mother-to-child transmission.

**Table 4:** Characteristics of quantitative studies included

#	Author, year	Country, setting	Study design	Participants	Sample size*	Outcome**
17	Albrecht 2006	Zambia, Urban	Clinical trial; sub-analysis	HIV+ women enrolled into the trial at two ANC clinics	760	Maternal/infant non-adherence (no ingestion of NVP)
18	Barigye, 2010	Uganda, Rural	Prospective cohort study	HIV+ women enrolled in PMTCT programme at four clinics	102	Receipt of maternal NVP; maternal/infant NVP ingestion
19	Delva, 2010	Kenya, Urban and rural	Prospective cohort study	Pregnant women attending ANC at five health centres	Not clear	Provision of NVP (defined as receipt of NVP)
20	Delvaux, 2009	Rwanda, Urban and rural	Case-control study	HIV+ women who did not adhere (cases)/adhered (controls) to PMTCT prophylaxis	236	Receipt of NVP; NVP adherence (ingestion in recommended time) in mothers and/or infants
21	Ekouevi, 2004	Cote d'Ivoire, Urban	Analysis within cohort study	Subset of HIV+ pregnant women within cohort study	1023	Women who started the prophylaxis regimen
22	Farquhar 2004	Kenya, Urban	Prospective cohort study	Pregnant women attending one clinic; male partners	314	Maternal receipt of NVP; maternal/infant dose administered
23	Karcher, 2006	Tanzania/Uganda, Rural	Prospective cohort study	Subset of HIV+ pregnant women attending four PMTCT sites	619	Infant NVP intake (administration)
24	Killam, 2010	Zambia, Urban	Intervention study; stepped-wedge design	HIV+ pregnant women at eight ANC clinics, eligible for cART	1566	Enrolment and initiation onto cART within 60 days of HIV diagnosis
25	Kinuthia, 2011	Kenya, Urban	Cross-sectional study	Subset of HIV+ women and their infants attending six MCH clinics	336	Mother and/or infant receipt of, or adherence to PMTCT ARVs
26	Kirsten, 2011	Tanzania, Rural	Prospective cohort study	HIV+ pregnant women enrolled in PMTCT programme at one site	122	Non-acceptance of, or adherence to prophylaxis
27	Kuonza, 2010	Zimbabwe Urban	Cross-sectional study	HIV+ pregnant women and their infants enrolled in PMTCT programme in four facilities	212	Maternal/infant non-adherence to NVP (no ingestion; ingestion >72 hrs post-birth or <2 hrs pre-delivery)
28	Megazzini 2009	Zambia, Urban	Clinical trial; sub-analysis	Pregnant women in the trial intervention arm who had HCT	71	Ingestion of NVP or calcium tablet >2/>1 hour before delivery
29	Mirkuzie, 2011	Ethiopia, Urban	Prospective cohort study	HIV+ women attending 15 facilities and their infants	219	Mother and/or infant receipt or ingestion of drugs
30	Msuya, 2008	Tanzania, Urban	Prospective cohort study	HIV+ pregnant women attending ANC at two public clinics	184	Maternal ingestion of NVP
31	Peltzer, 2008	South Africa, Unclear	Cross-sectional study	HIV+ pregnant women in a PMTCT cohort from five clinics	116	Maternal/infant adherence to NVP (consumption)
32	Peltzer, 2010	South Africa, Rural	Cross-sectional study	Post-natal HIV+ women and their infants at 47 clinics	815	Mother and/or infant not ingesting NVP, or not at recommended time
33	Peltzer, 2011	South Africa, Rural	Cross-sectional study	HIV+ pregnant/post-natal women and their infants at 48 clinics	746	Maternal/infant adherence to ARV prophylaxis (NVP- ingestion; AZT-never missed dose)
34	Stinson, 2010	South Africa, Urban	Retrospective cohort study	HIV+ women eligible for cART attending four ANCs	516	Initiating cART during pregnancy; on cART at delivery
35	Stringer, 2003	Zambia, Urban	Cluster-randomized trial	HIV+ pregnant women attending the two health facilities in the trial	201	Maternal ingestion of NVP
36	Stringer, 2010	Four countries, Unclear	Cross-sectional study	HIV+ women and their infants attending 43 delivery sites	3196	Maternal/infant NVP ingestion

* Sample size for analysis associated with uptake of ARVs

** Some studies also analyzed other outcomes that are not shown.

# Study number (sequential order; differs from bibliographic reference number); VCT=voluntary counselling and testing; cART=combination antiretroviral therapy; NVP=nevirapine; AZT=Azidothymidine; ANC=antenatal clinic; MCH=maternal and child health; PMTCT=Prevention of mother-to-child transmission; HCT=HIV counselling and testing.

**Table 5:** Characteristics of mixed-methods studies included

#	Author, year	Setting	Study design	Participants	Sample size*	Outcomes** (quantitative)
37	Balcha, 2011	Ethiopia, urban and rural	IDIs/descriptive analysis of aggregated programme data	IDIs with key informants	3 IDIs	Uptake of PMTCT indicators only
38	Doherty 2009	South Africa, rural	Operational research: FGDs, observations, structured interviews, descriptive analysis of routine PMTCT data	Facility managers, counsellors, primary health care supervisors, district coordinators	15 interviews with managers/ 35 with counsellors; 1 FGD	Uptake of PMTCT indicators only
39	Kiarie, 2003	Kenya, urban	FGDs/randomized clinical trial	HIV+ postpartum/ pregnant women	124 (quantitative analysis); 7 FGDs	Compliance: took maternal and infant NVPor≥80% of AZT doses
40	Laher, 2012	South Africa, urban	Cross-sectional survey/structured interviews and FGD	Women attending a paediatric clinic with HIV-infected infants	Survey: 45; 2 FGDs: 10 women in total; Interviews: 35	Uptake of PMTCT indicators only
41	Mepham, 2011	South Africa, rural	IDIs/quantitative sub-study within clinical trial	Subset of HIV+ women enrolled into the trial	94 (quantitative analysis); 43 IDI	No statistical analysis of factors associated with PMTCT ARV uptake
42	Muchedzi, 2010	Zimbabwe urban	FGDs/cross-sectional study	HIV+ women from 4 ANCs referred for cART and key informants (from ANC)	Survey: 147; 2 FGDs (of 10–12)	Registration at the HIV clinic
43	Varga, 2008	South Africa, urban and rural	Participatory group workshops (role plays), FGDs and cross-sectional survey	RCH clinic/programme staff and adolescent mothers	10–15 per workshop (×2); 10–12 per FGD (×2); 100 for survey	No statistical analysis of factors associated with PMTCT ARV uptake
44	Watson-Jones, 2012	Tanzania, urban	Cohort study/structured interviews and observations	HIV+ women at 2 delivery wards/health workers	Cohort analysis: 175; Observations: 9; IDI sample unclear	Attendance at the HIV clinic up to 4 months post-delivery

* Sample size for qualitative work and/or quantitative analysis associated with uptake of ARVs.

** Some studies also analyzed other quantitative outcomes that are not shown; quantitative analyses for study numbers 37, 38, 40, 41 and 43 were excluded (only qualitative component met inclusion criteria).

# Study number (sequential order; differs from bibliographic reference number); cART=combination antiretroviral therapy; NVP=nevirapine; AZT=Azidothymidine; ANC=antenatal clinic; MCH=maternal and child health; PMTCT=Prevention of mother-to-child transmission; IDI=In-depth interview; FGD=Focus group discussion.

### Quality appraisal results

Most qualitative studies (15/16) were appraised as good quality (scored≥10 on the checklist) (supplement 2a). Six qualitative components of mixed-methods designs (out of eight) scored below 10, where methods were brief or the research was operational. Authors rarely (2/24 studies) acknowledged their own role in influencing the research. Analysis by ≥2 assessors was infrequently mentioned (7/24 studies). The sensitivity analysis did not reveal any major changes in results or interpretation: the main themes remained the same, in broadly the same relative order of importance.

Twenty quantitative studies were excluded from this review because they did not report a multivariate analysis, or had unclear statistical methods (*n*=5). Included quantitative research was mostly good quality (scored≥10) (supplement 2b), with only one paper excluded in the sensitivity analysis; this did not result in changes in conclusions. Small sample sizes (for example sub-analyses) and potential lack of power was a common issue, but not often discussed by authors. Residual confounding was possible in at least 1/3 of the studies.

### Barriers and facilitating factors to uptake of ARVs for PMTCT

Barriers and facilitating factors fell into three broad categories relating to individuals, their partners and community, and health systems. This hierarchy, described as a socio-ecological model, has previously been applied to PMTCT and HIV health-services research [15, 26]. Findings are summarized in Tables 6 and 7.

**Table 6:** Factors associated with PMTCT ARV uptake in the included qualitative research

	Study number																							
	
Factors	1	2	3	4*	5	6	7	8	9	10	11	12	13	14	15	16	37*	38*	39*	40	41*	43	44*	Total
**Individual**																								
***Psychological***		✓	✓	✓			✓		✓		✓		✓	✓					✓	✓				10
Denial/shock (following results)/depression		✓	✓	✓			✓		✓		✓		✓	✓					✓					9
Fear (of being HIV positive/death/ARVs)		✓							✓				✓							✓				4
Desire to protect baby/self/family (facilitating)													✓	✓										2
Feeling better, well after taking cART (facilitating)														✓										1
***Knowledge and beliefs***		✓		✓	✓	✓		✓			✓		✓		✓				✓		✓			10
Poor knowledge of HIV/MTCT/ARVs		✓		✓	✓	✓		✓			✓		✓		✓				✓		✓			10
Scepticism about ARVs						✓		✓			✓		✓		✓				✓					6
***Obstetric factors and pregnancy-history***							✓		✓	✓		✓							✓					5
Sudden/unclear/early/night-time onset of labour							✓		✓	✓		✓							✓					5
Post-delivery ill-health										✓														1
***Disease progression***					✓			✓						✓										3
Lack of symptoms – (perceived) disease severity					✓			✓						✓										3
***Personal management and supply of treatment***				✓					✓	✓			✓						✓	✓	✓			7
Lost/sold/stolen/forgetting/ran out of tablets				✓					✓	✓									✓		✓			5
Difficulties administering infant treatment													✓							✓				2
**Partner and Community**																								
***Stigma***	✓	✓	✓	✓	✓	✓	✓			✓	✓	✓	✓	✓		✓	✓		✓	✓	✓	✓		18
***Disclosure issues*/*fear of disclosure***	✓		✓	✓	✓	✓	✓			✓	✓	✓	✓	✓		✓	✓		✓	✓	✓	✓		17
Relationship strains/violence	✓					✓				✓			✓	✓							✓			6
Fear of someone finding/seeing pills	✓					✓						✓									✓			4
Partners controlling finances					✓									✓										2
***(Lack of) community*/*relative*/*partner support***	✓	✓	✓	✓	✓		✓			✓			✓	✓			✓							10
Unwillingness of partners to test					✓									✓										2
Partner support (facilitating)	✓													✓										2
***Cultural traditions and beliefs***	✓	✓					✓			✓			✓	✓	✓		✓				✓			9
Preference for TBAs/home-births	✓	✓					✓			✓					✓									5
Traditional medicines/healers	✓												✓	✓							✓			4
Strong role of grandparents, associated beliefs	✓									✓														2
Scepticism regarding facilities in general															✓		✓							2
**Health-systems**																								
***Client–staff interactions***		✓	✓		✓			✓	✓	✓	✓	✓		✓		✓			✓			✓		12
Staff attitudes/fear of negative attitudes		✓	✓		✓				✓	✓	✓	✓		✓		✓			✓			✓		11
Trust in staff/helpful advice/support (facilitating)			✓							✓		✓		✓										4
Fear of lack of confidentiality																						✓		1
Health-worker–client power imbalance								✓														✓		2
***Resources and infrastructure***		✓	✓	✓	✓			✓	✓		✓	✓		✓		✓	✓	✓	✓			✓	✓	15
Staff shortages			✓	✓	✓			✓	✓		✓	✓		✓		✓	✓	✓					✓	12
Long waiting times			✓		✓			✓			✓			✓		✓								6
Staff too busy/workload high/stressed			✓	✓				✓			✓							✓				✓	✓	7
Lack of training/trained staff				✓																				1
Counselling sessions too short/too few								✓																1
Staff failings									✓									✓					✓	3
Failure to give NVP/poor instructions									✓															1
Late bookings for delivery																		✓						1
Misunderstanding of client services required																							✓	1
Drug or supplies shortages								✓	✓			✓					✓	✓						5
Delays (HIV tests, results, CD4 counts)									✓			✓					✓							3
Privacy issues (layout)			✓	✓										✓		✓		✓	✓					6
Integration of services		✓						✓								✓							✓	4
Poor referral links/no/delayed referral to cART								✓															✓	2
Integration as a facilitating factor		✓														✓								2
Poor coordination between regional/local levels		✓															✓							2
Poor record keeping												✓												1
***Access to facilities*/*services***		✓	✓		✓		✓	✓		✓	✓			✓			✓		✓					10
Transport issues/time and cost		✓	✓		✓		✓	✓		✓	✓			✓			✓		✓					10
Costs/perceived costs of services/treatment		✓					✓	✓			✓													4
***Late first presentation to ANC***													✓							✓				2

ANC=antenatal clinic; ARV=antiretroviral; cART=combination antiretroviral therapy; MTCT=mother-to-child transmission; NVP=nevirapine; PMTCT=prevention of mother-to-child transmission; TBA=traditional birth attendant.

✓ indicates the factor was related to PMTCT ARV uptake

* Studies removed during sensitivity analysis of qualitative results.

**Table 7:** Factors associated with PMTCT ARV uptake in the included quantitative research, and cases where these factors were explored but no statistical evidence for an association with PMTCT ARV uptake was reported

	Study number																								
	
Factors	17	18	19	20	21	22	23*	24	25	26	27	28	29	30	31	32	33	34	35	36	39	42	44	Total	
**Individual**																									
***Socio-demographic***																									
Education (or literacy)	✓	✓		✓	✓		✓		×	×	✓		×		×	×	×(u)		✓		×	×	×	16	
Age of mother	×	✓		×			✓		×	✓			×		×	×		×	×	✓	×	×	×	15	
Religion				×			✓				×(u)												×	4	
Ethnicity																							✓	1	
***Socio-economic***																									
Income activities/occupation	×				×		×		×	✓	×								×			×	×	9	
***Knowledge and beliefs***																									
HIV/MTCT knowledge				×											×	✓	×(u)				×(u)	×		6	
Lived in villages exposed to HIV research		✓																						1	
***Obstetric and pregnancy-history***																									
Mother took PMTCT prophylaxis				✓																			✓	2	
PMTCT in previous pregnancy											✓													1	
Parity	×						×		×		✓								×(u)					5	
Cervical dilation												✓												1	
Term/premature delivery																✓								1	
Caesarian/vaginal delivery																×				✓				2	
***Infant factors/characteristics***																									
Birth weight of infant	✓																			✓				2	
Knowledge of infant HIV status																✓								1	
At risk for neonatal death	✓																							1	
**Partners and community**																									
***Stigma***																									
Internalized stigma									✓								×(u)							2	
Experience of HIV/AIDS discrimination																×(u)	✓					×		3	
***Disclosure***																									
Disclosure of HIV/ARVs to partner	✓			×(u)					×		✓		×		✓	✓			×		×(u)			9	
Disclosure to anyone		×		×						×(u)					×(u)	×(u)	✓						✓	7	
Disclosure to other (not partner)				✓																				1	
***Married or living with partner***		×		✓	✓				×	×						×	×(u)		×		×	✓	×	11	
***Support***																									
Partner VCT		×		✓										×(u)		✓					×(u)		×	6	
Couples VCT	×					✓																		2	
Male involvement																×	✓							2	
Attendance at support group																×(u)	×(u)					✓		3	
**Health-systems**																									
ARV services integrated into ANC								✓										×						2	
Client understood referral process																						✓		1	
HIV status kept confidential at clinic																✓								1	
Site of PMTCT counselling							✓																	1	
Place of delivery	✓	×		×(u)					✓		✓		✓		×	×(u)					✓			9	
Urban/rural facility			✓																					1	
Number of ANC visits				✓							×(u)	✓			×	×(u)				✓			×(u)	7	
Gestational age at first ANC visit		✓		×(u)						✓			×					✓						5	
HIV test after/at first ANC visit				✓																				1	
Mother given NVP to take home											✓													1	
Regimen type													×							✓	✓			3	
Universal NVP without HIV testing																			✓					1	

✓ indicates statistical evidence for an association (*p*<0.05 or 95% CI excludes the null value of one) was reported with at least one relevant outcome (adherence/receipt of PMTCT ARVs/cART/attendance at ART clinic).

× Indicates no statistical evidence for an association

×(u) Statistical evidence for an association in uni-variate analysis only.

ANC=antenatal clinic; ARV=antiretrovirals; MTCT=mother-to-child transmission; NVP=nevirapine; PMTCT=Prevention of mother-to-child transmission; VCT=voluntary counselling and testing; ART=Antiretroviral therapy.

* Study removed during sensitivity analysis of quantitative results.

#### Individuals

##### Socio-demographic factors

Maternal education and age were the most frequently investigated factors in quantitative analyses. Seven studies reported an association between lower maternal educational level/literacy and not receiving/taking ARV prophylaxis [27–33]. Nine quantitative studies investigated maternal education but found no association [34–42], and were conducted more recently (7/9 since 2008) than the studies reporting associations (5/7 prior to 2007).

The majority of studies (11/15) exploring maternal age found no association with uptake of PMTCT ARVs [27, 29, 33, 34, 36–38, 40–43], although four found that younger mothers (<20–25 years) were less likely to receive/adhere to prophylaxis [35, 44], or to receive NVP for their infants (cross-regional, Tanzanian and Ugandan studies) [28, 31]. Mixed-methods research in South Africa also highlighted difficulties and discrimination faced by adolescents participating in PMTCT services [45].

##### Knowledge and individual beliefs

Poor knowledge of HIV transmission and ARV drugs emerged frequently as a reason for dropping out of PMTCT programmes and failing to access/ingest ARVs in qualitative research [38, 41, 46–53]. Doubts about the efficacy of ARVs for MTCT [41, 49, 51, 52] and beliefs that ARVs could cause HIV [50] or harm the unborn child [54] were raised. In quantitative research in rural South Africa, women with higher PMTCT knowledge scores were more likely to adhere to NVP [38], though there was no/weak evidence for an association between knowledge scores and adherence to prophylaxis in other Southern and East African studies [29, 37, 39, 41, 42].

##### Psychological factors

Qualitative work revealed psychological barriers to initiating and adhering to PMTCT interventions following an HIV diagnosis. Women described shock, depression and denial on learning their status at antenatal clinics (ANC) [41, 51, 54–59], as well as fears about their condition and death [47, 58], or handling side effects and lifelong treatment [54].

Regaining health in response to cART, and a mother's desire to protect her own/unborn baby's health and to care for her family, were facilitating factors for initiating/continuing with combination treatment [54, 59].

##### Obstetric and pregnancy-history factors

Qualitative studies highlighted the difficulties that rural women faced in reaching the clinic following sudden onset of labour, particularly at night [41, 57, 58, 60]. In quantitative studies, greater cervical dilation on admission (reflecting late admission to the delivery ward or rapid labour) [61] and pre-term deliveries/low infant birth weight were associated with lower odds of ingesting treatment [27, 38, 44]. Ill-health following home-delivery prevented/delayed women from taking their baby to the facility for prophylaxis [60]. Poor infant health was also associated with the infant not receiving NVP [27]. Maternal adherence to PMTCT ARVs influenced subsequent adherence to prophylaxis by the newborn [29] and linkage to HIV care and treatment [40].

##### Disease progression

Three qualitative studies revealed that HIV-positive pregnant women lacking symptoms did not feel they needed ARVs for PMTCT [48, 52, 59]. However, immunological status (CD4 count) was not significantly associated with adherence to NVP in quantitative analyses [27, 33, 35, 36].

##### Personal management and supply of treatment

Losing or selling tablets, forgetting to take them, running out, or finding them stolen affected ARV adherence in qualitative research [41, 46, 53, 58, 60], as well as difficulties administering infant prophylaxis due to tolerability issues (e.g. vomiting) [62].

##### Partners and community

###### Stigma, disclosure of HIV status and community support

Stigma regarding HIV status and fear of disclosure to partners or family members (particularly grandmothers or mothers-in-law) were major barriers to uptake of PMTCT ARV interventions in almost all of the qualitative research [41, 45–49, 51, 53–57, 59, 60, 62–66]. Two quantitative studies reported associations between stigma measures and PMTCT ARV use [34, 38], including self-stigma (also mentioned in qualitative research [51, 54, 57, 64]). Discrimination directed specifically at pregnant HIV-positive women (blame for potentially dying and leaving an orphaned baby) was described in one qualitative study in Kenya [64].

In quantitative studies, non-disclosure of HIV status to partners, or not telling them about NVP, was associated with not attending the HIV clinic in Tanzania [40], and not ingesting maternal or infant prophylaxis in South Africa [37, 38], Zimbabwe [32] and Zambia (among home-births [27]). Similarly, married women or those living with a male partner were less likely to use prophylaxis or access cART in three studies [29, 30, 42], although there was no evidence for an association in other analyses [28, 33–35, 38–41].

Qualitative research confirmed that fear of disclosure could deter HIV-positive women from attending HIV clinics and initiating treatment [45, 49, 51, 54, 55, 59, 66], from ingesting or storing ARVs [46, 48, 57, 60, 62], or from seeking/administering infant prophylaxis [53, 57, 64]. Some women faced or feared negative reactions from their partners including refusals to test for HIV, abandonment or violence [48, 49, 54, 59, 60, 67]. Conversely, women who did not disclose their status were more likely to take their medication in one qualitative study (these women were better accepted by their community and life could continue as normal, while those whose positive status was known faced stigmatization) [64].

Lack of partner or family support was frequently mentioned [47, 48, 53–55, 57, 59, 60, 65], while support was also a facilitating factor [42, 57, 59, 60, 64]. Partner/couples voluntary counselling and testing (CVCT) was related to elevated adherence to/receipt of prophylaxis in three quantitative studies [29, 38, 67], although five studies found no/weak evidence for an association [27, 28, 40, 41, 68].

###### Cultural traditions

General scepticism towards facilities or modern medicine among community or family members, and strong roles of elders and their beliefs, could influence decisions to use traditional healers and medicines alongside/in place of ARVs [46, 54, 59, 64], as well as place of delivery [preferences for traditional birth attendants (TBAs) and home-births] [47, 50, 57, 60, 64].

#### Health systems Resources and infrastructure

Shortage of (trained) clinic staff was a major barrier to PMTCT ARV uptake [48, 51–53, 55, 58, 59, 63, 65, 66, 69]. As a result, health workers were overwhelmed with high patient volume, contributing to long waiting-times [40, 48, 51, 52, 55, 59, 66], brief or poor quality counselling sessions [52], staff stress [45, 52], staff failings and misunderstandings by staff [40, 58].

Shortages of resources (including ARVs) [52, 58, 63, 65, 69], poor record keeping [63], and poor integration of services, referral links or tracking systems [40, 47, 52, 66], also contributed to low uptake of ARVs. Integrated ANC and ARV services (ARV drugs provided within the ANC building) was related to improved uptake of cART or prophylaxis in quantitative [70] and qualitative research [47, 66], although uptake was comparable if cART services were offered one day per week at ANC or in separate buildings/sites in another quantitative study [43].

Staff-client interactionsExperience of negative staff attitudes was a frequently cited barrier to returning to facilities [41, 45, 47, 48, 51, 55, 58, 60, 63, 66], limiting the opportunity to receive prophylaxis or cART [48]. For example, women experienced or feared scolding from staff for home-deliveries when returning with their baby for NVP administration [60], or were stigmatized [45, 48]. Confidentiality breaches were reported in one study [45], and sub-optimal layout of clinics contributed to lack of privacy [41, 53, 55, 59, 69]. However, some women described how counsellors helped them to persevere with PMTCT interventions or HIV clinic attendance, and deal with stigma, disclosure and relationship issues [59, 63].

Access to servicesAnother key issue affecting access to PMTCT treatment for mothers and infants was the distance to facilities and frequency of visits required [41, 47, 48, 51, 52, 55, 57, 59, 60, 65], particularly in rural areas [57, 60, 65]. Perceived or real costs of maternity services and treatment were sometimes concerns [47, 51, 52, 57].

Home-births resulted in barriers to mothers and infants receiving PMTCT ARVs in quantitative [32, 34, 36, 41] and qualitative studies [47, 50, 57, 60] conducted in rural and urban settings. For example, distance or fear of inadvertent disclosure hindered women from returning to facilities for infant prophylaxis [57, 60]. In some settings, women were provided with NVP (to take during labour, and/or give to their infant) during ANC appointments, resulting in improved PMTCT ARV ingestion in mother-baby pairs in one quantitative study [32] and no association between place of delivery and uptake of prophylaxis in three other quantitative studies [28, 29, 37].

Late presentation at ANC was a barrier to accessing ARVs in two qualitative studies [54, 62]. Similarly, one quantitative study suggested earlier enrolment at ANC was associated with better uptake of cART during pregnancy [43]. However, other quantitative research found women presenting earlier in pregnancy were less likely to take NVP [28] or collect AZT prescriptions [35].

Type of ARV regimen taken during pregnancy influenced maternal adherence: women taking twice-daily AZT were less likely to adhere than women taking NVP [41], while Stringer *et al*. found that women on cART were more likely to adhere than those taking NVP alone [44].

### Changing factors over time

Dividing the studies into those conducted pre- and post-2007 revealed no major difference in community and health-systems factors over time (Tables 8 and 9). Psychological factors (denial/shock/depression), knowledge and maternal education were less frequently reported in recent studies (compared to earlier studies). It would have been preferable to take into account the number of studies that had *investigated* each factor within each time period, however details of the topic guides were not often provided in the manuscripts of qualitative studies, and not all quantitative study manuscripts clearly listed all the variables that were collected and analyzed.

**Table 8:** Changes over time: factors associated with PMTCT ARV uptake in qualitative research

	Number of studies (%)			
	
Factors	Fieldwork before 2007* (N=12)		Fieldwork in/after 2007** (N=11)	
**Individual**				
***Psychological***	7	58%	3	27%
Denial/shock (following results)/depression	7	58%	2	18%
Fear (of being HIV positive/death/ARVs)	2	17%	2	18%
Desire to protect baby/self/family (facilitating)	0	0%	2	18%
Feeling better, well after taking cART (facilitating)	0	0%	1	9%
***Knowledge and beliefs***	8	67%	2	18%
Poor knowledge of HIV/MTCT/ARVs	8	67%	2	18%
Scepticism about ARVs	5	42%	1	9%
***Obstetric factors and pregnancy-history***	3	25%	2	18%
Sudden/unclear/early/night-time onset of labour	3	25%	2	18%
Post-delivery ill-health	0	0%	1	9%
***Disease progression***	2	17%	1	9%
Lack of symptoms – (perceived) disease severity	2	17%	1	9%
***Personal management and supply of treatment***	3	25%	4	36%
Lost/sold/stolen/forgetting/ran out of tablets	3	25%	2	18%
Difficulties administering infant treatment	0	0%	2	18%
**Partner and Community**				
***Stigma***	9	75%	9	82%
***Disclosure issues/fear of disclosure***	8	67%	9	82%
Relationship strains/violence	1	8%	5	45%
Fear of someone finding/seeing pills	1	8%	3	27%
Partners controlling finances	1	8%	1	9%
***(Lack of) community/relative/partner support***	5	42%	5	45%
Unwillingness of partners to test	1	8%	1	9%
Partner support (facilitating)	0	0%	2	18%
***Cultural traditions and beliefs***	3	25%	6	55%
Preference for TBAs/home-births	3	25%	2	18%
Traditional medicines/healers	0	0%	4	36%
Strong role of grandparents, associated beliefs	0	0%	2	18%
Scepticism regarding facilities in general	1	8%	1	9%
**Health-systems**				
***Client–staff interactions***	8	67%	4	36%
Staff attitudes/fear of negative attitudes	7	58%	4	36%
Trust in staff/helpful advice/support (facilitating)	1	8%	3	27%
Fear of lack of confidentiality	1	8%	0	0%
Health-worker–client power imbalance	2	17%	0	0%
***Resources and infrastructure***	9	75%	6	55%
Staff shortages	6	50%	6	55%
Long waiting times	4	33%	2	18%
Staff too busy/workload high/stressed	5	42%	2	18%
Lack of training/trained staff	1	8%	0	0%
Counselling sessions too short/too few	1	8%	0	0%
Staff failings	1	8%	2	18%
Failure to give NVP/poor instructions	1	8%	0	0%
Late bookings for delivery	0	0%	1	9%
Misunderstanding of client services required	0	0%	1	9%
Drug or supplies shortages	2	17%	3	27%
Delays (HIV tests, results, CD4 counts)	1	8%	2	18%
Privacy issues (layout)	3	25%	3	27%
Integration of services	2	17%	2	18%
Poor referral links/no/delayed referral to cART	1	8%	1	9%
Integration as a facilitating factor	0	0%	1	9%
Poor coordination between regional/local levels	1	8%	1	9%
Poor record keeping	0	0%	1	9%
***Access to facilities/services***	7	58%	5	45%
Transport issues/time and cost	7	58%	3	27%
Costs/perceived costs of services/treatment	3	25%	0	0%
***Late first presentation to ANC***	0	0%	2	18%

* Study numbers and fieldwork dates: 2 (2001), 3 (2005), 4 (2003), 5 (2006), 6 (2006), 7 (2006), 8 (2009), 9 (2005), 11 (1998–99), 15 (2006), 39 (1999–2001), 43 (2002–3).

** Study numbers and fieldwork dates: 1 (2010—imputed one year before year of publication), 10 (2008), 12 (2008–9), 13 (2007–8), 14 (2009–10), 38 (2007), 37 (2007–8), 40 (2009), 41 (2008), 44 (2008–9).

**Table 9:** Changes over time: factors associated with PMTCT ARV uptake in quantitative research

	Number of studies (%)			
	
Factors	Fieldwork before 2007* (N=13)		Fieldwork in/after 2007** (N=10)	
**Individual**				
***Socio-demographic***				
Education (or literacy)	6	46%	1	10%
Age of mother	2	15%	2	20%
Religion	1	8%	0	0%
Ethnicity	0	0%	1	10%
***Socio-economic***				
No income generating activity	0	0%	1	10%
***Knowledge and beliefs***				
HIV/MTCT knowledge	0	0%	1	10%
Lived in villages exposed to HIV research	1	8%	0	0%
***Obstetric and pregnancy-history***				
Mother took PMTCT prophylaxis	1	8%	1	10%
PMTCT in previous pregnancy	0	0%	1	10%
Parity	0	0%	1	10%
Cervical dilation	1	8%	0	0%
Term/premature delivery	0	0%	1	10%
Caesarian/vaginal delivery	0	0%	1	10%
***Infant factors/characteristics***				
Birth weight of infant	1	8%	1	10%
Knowledge of infant HIV status	0	0%	1	10%
At risk for neonatal death	1	8%	0	0%
**Partners and community**				
***Stigma***	0	0%	0	0%
Internalized stigma	0	0%	1	10%
Experience of HIV discrimination	0	0%	1	10%
***Disclosure***				
Disclosure of HIV/ARVs to partner	2	15%	2	20%
Disclosure to anyone	0	0%	2	20%
Disclosure to other (not partner)	1	8%	0	0%
***Married or living with partner***	2	15%	1	10%
***Support***				
Partner VCT	1	8%	1	10%
Couples VCT	1	8%	0	0%
Male involvement	0	0%	1	10%
Attendance at support group	0	0%	1	10%
**Health-systems**				
ARV services integrated into ANC	0	0%	1	10%
Client understood referral process	0	0%	1	10%
HIV status kept confidential at clinic	0	0%	1	10%
Site of PMTCT counselling	1	8%	0	0%
Place of delivery	2	15%	3	30%
Urban/rural facility	1	8%	0	0%
Number of ANC visits	2	15%	1	10%
Gestational age at first ANC visit	2	15%	1	10%
HIV test after/at first ANC visit	1	8%	0	0%
Mother given NVP to take home	0	0%	1	10%
Regimen type	1	8%	1	10%
Universal NVP without HIV testing	1	8%	0	0%

* Study numbers and fieldwork dates: 17 (2001–3), 18 (2002–7), 19 (2004–6), 20 (2006), 21 (2000–2), 22 (2001–2), 23 (2002–4), 28 (2005–6), 30 (2002–4), 34 (2005), 35 (2000–1), 39 (1999–2001).

** Study numbers and fieldwork dates: 24 (2007–8), 25 (2008–9), 26 (2008–9), 27 (2008), 29 (2009), 31 (2005–6), 32 (2008–9), 33 (2010—imputed one year before year of publication), 36 (2007–8), 42 (2008), 44 (2008–9).

## Discussion

### Main findings

This is the first study, to our knowledge, to systematically review barriers and facilitating factors to uptake of antiretrovirals (both prophylaxis and cART) for PMTCT in sub-Saharan Africa, from both a qualitative and quantitative perspective, thus allowing an analysis of the relative importance of different factors and changes over time. The identified factors fell broadly into individual, community and health-systems levels.

At the individual-level, poor knowledge of HIV/MTCT/ARVs, lower maternal educational level (potentially manifested through poor knowledge, or differences in socio-economic status), and psychological factors following diagnosis of HIV were the key barriers that emerged from the review. Stigma and fear of disclosure to partners/others were the most frequently cited barriers overall, with stigmatization occurring at all levels (self-stigma, discrimination by partners, community members and health workers). The extent of community/partner support was another major factor affecting uptake of PMTCT ARVs, while cultural traditions including preferences for traditional healers and TBAs were also common. Key health-systems barriers were staff shortages, (fear of) scolding from staff, facility accessibility issues and non-facility deliveries.

Qualitative results provided arguably the most useful insights, for example describing barriers and facilitating factors from the perspective of the patient or provider and *how* they affected ARV uptake. However, few qualitative studies ranked barriers in terms of relative importance, which would facilitate interpretation and help to prioritize intervention strategies. Quantitative results were less conclusive due to heterogeneity and the diversity of factors investigated. Some important factors that are difficult to measure, such as stigma, were not often investigated in quantitative work, while socio-demographic factors that are more easily measured were frequently analyzed. Therefore, the frequency of citations for a particular factor does not necessarily reflect its importance, but the number of times it has been investigated.

### Programmatic implications

**Figure 3:**
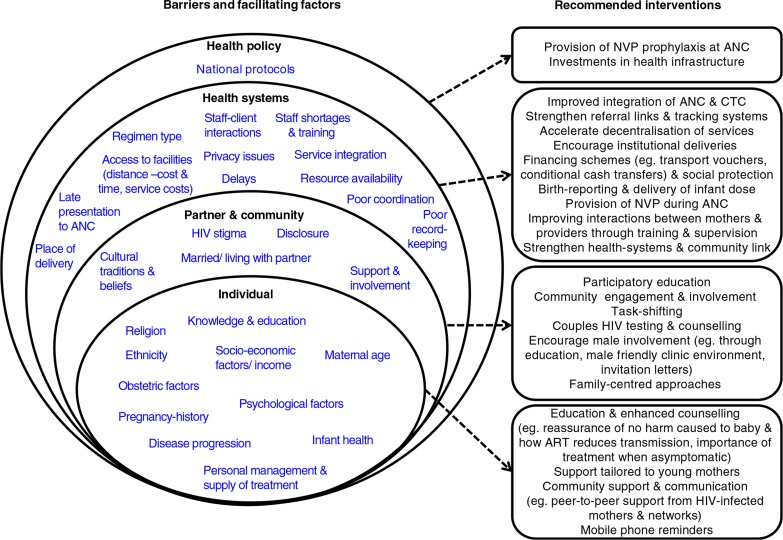
Factors affecting uptake of ARVs for PMTCT identified in the literature review are populated within a hierarchy of individuals (pregnant women or infants), their community and health systems around them, which are in turn part of the wider health-policy environment. A complex interplay of factors from each level ultimately impacts on PMTCT ARV uptake. This hierarchy is adapted from a socio-ecological model [72]. Possible interventions and policy recommendations addressing barriers at each level are illustrated to the right-hand side. Some interventions may address more than one barrier within a level, or barriers at multiple levels, and may be packaged together. ART=Antiretroviral therapy; ANC=antenatal clinic; CTC=(HIV) Care and treatment clinic; NVP=nevirapine.

Addressing barriers at each social/structural level is essential in order to realize the full potential of PMTCT programmes. Mitigating these barriers may also have benefits beyond improved uptake of PMTCT programmes, in reducing the risk of transmission to sero-discordant partners of HIV-positive mothers [71]. Figure 3 illustrates recommended interventions to address barriers at different levels.

At the individual-level, poor knowledge could be addressed through counselling and educational strategies. However, authors occasionally noted educational efforts had made little impact on service use. Delva *et al*. suggested transport fares or experience from previous pregnancies may have been a greater barrier to accessing services [56]. Careful distribution of information will also be needed, as attempts to educate women using pamphlets have occasionally failed due to illiteracy or fear of disclosure (being seen with the documents) [48]. Further counselling by health-providers (for example providing reassurance that ARV treatment will not harm the unborn baby and explaining in simple terms how the drugs can reduce transmission) could encourage women to take their treatments, as well as community-driven participatory communication strategies [73], or new technologies (such as mobile phone reminders) [74].

The frequency of citations of psychological barriers (denial, depression and fears about being HIV-positive, treatment and death) in the qualitative research, also suggests the need for strengthened supportive counselling. Careful management and advice for women initiating ARVs early in pregnancy will be needed, to ensure they do not discontinue treatment if symptoms decrease. Peer-support from other HIV-positive individuals, for example Mothers2mothers and “networks” programmes may also offer psychological support [73, 75–77]. Support tailored for pregnant adolescents could also help address their fears and other social and legal challenges faced by this particularly vulnerable group. Barriers to PMTCT ARV uptake were not explored among any other specific populations (such as sex-workers or refugees), representing an area for further research.

The prominence of community-level barriers, particularly stigma and fear of disclosure, suggests the need for approaches that engage communities and create an enabling environment for PMTCT. Further education about PMTCT and sensitization to HIV, particularly focussing on HIV infection during pregnancy, is needed to reduce stigma and improve disclosure. Approaches might include participatory educational group activities [78–80], and involvement of HIV-positive individuals in tackling stigma, offering peer-support and counselling [75].

The effect of disclosure may be context/culture-specific (possibly explaining heterogeneity in findings for this factor). Although qualitative findings suggested overwhelmingly that fear/lack of disclosure hindered uptake of PMTCT ARVs, women living in rural Kenya who concealed their HIV status in order to preserve family stability, follow traditions and please elders were more likely to adhere to PMTCT services [64]. This suggests community-level approaches including elders and community leaders, and solutions tailored to the setting, are needed. Such approaches might also address preferences for traditional healers and TBAs.

Tackling disclosure issues may benefit uptake of infant prophylaxis, as non-disclosure appeared to be an important factor limiting receipt of NVP among infants in qualitative and quantitative research, particularly in the case of home-births [29, 32, 37, 38, 53, 57, 64]. However, a minority of studies specifically reported barriers to receiving the infant dose (quantitative studies often combined maternal and infant outcomes), suggesting a need for further research.

Lack of support (emotional, financial or physical) was another frequently cited barrier to PMTCT ARV uptake, and good support was occasionally noted to improve uptake in qualitative and quantitative research. Support and disclosure are likely to become increasingly important in the context of “Option B+.”

Several authors suggested engaging men in the PMTCT programme to improve communication, disclosure and support [47–49, 53, 55, 56, 67]. A review of family-centred approaches to PMTCT also described positive outcomes of partner participation at different points of the PMTCT cascade [81]. However, other studies did not support this finding [27, 28, 40, 41] and a Cochrane review (2012) concluded there was insufficient rigorous evidence for the effectiveness of male involvement on PMTCT services, highlighting that further evaluation is required. While partner VCT is advised in many national PMTCT guidelines, levels of male involvement were often very low, and few studies reported male perspectives on involvement in pregnancy/delivery. Research is emerging on how to implement CVCT/male involvement in the context of ANC and other settings, for example, through invitations given to partners and “male friendly” clinics including flexible opening hours and priority for couples [15, 67, 68, 82–84]. However, evidence for the effectiveness of these interventions remains limited. It is clear that while male involvement holds promise for improving uptake of PMTCT interventions, further rigorous evaluation and implementation research is needed.

Expanding PMTCT services to include other family members (for example in counselling) has also shown promise [81] and may be particularly important for single women or those with unsupportive partners.

At the health-systems level, staff shortages, (fear of) scolding from staff, and facility accessibility issues emerged frequently during qualitative investigations. Addressing these issues should be a priority. Community-based approaches may help to overcome the shortfalls in health workers through task-shifting [59, 85–87], although evaluation of these strategies in the context of PMTCT is limited and concepts are mostly inferred from HIV or maternal health programmes more broadly (reviewed by Buzsa *et al*. [15]).

Accelerating service decentralization, particularly to rural areas, financing schemes such as provision of transport and service vouchers [88], conditional cash-transfers [89], or transport services provided by community members [88, 90] may alleviate access issues. Provision of the NVP dose during ANC appointments, or birth-reporting strategies, have the potential to improve NVP uptake in mothers and/or infants and could be implemented more widely, particularly where a high proportion of women have home-births.

Improving interactions between mothers and health-providers, for example through toolkits, training and supervision should be promoted to allay fears of negative staff reactions and to capitalize on facilitating effects of trust in staff. Confidentiality and privacy issues could be addressed by optimizing facility layout. Participatory improvement approaches involving staff might also address poor behaviour, low morale and record keeping [69], while attention should also be given to underlying systemic issues such as sufficient and regular staff payments.

While supply shortages (of HIV test kits and drugs) were mentioned to a lesser extent than staff and accessibility issues, they were mostly noted by health-providers, so the relatively smaller number of citations may reflect that fewer studies included such participants. As the availability of working tools is a pre-requisite for a fully functioning PMTCT programme, this should be considered a critical issue.

Integration of ANC and cART services appeared to facilitate linkage of HIV-positive pregnant women to care and treatment [70], although partial integration seemed less effective [44] (reviewed by Ferguson *et al*. [14]), and a Cochrane review (2011) called for further research on the effect of integration of PMTCT with other health services due to paucity of data meeting inclusion criteria [91]. Retention after successful transition to an HIV clinic also requires further exploration.

### Changes over time

The majority of studies reviewed were conducted in early stages of PMTCT programmes prior to cART provision, so further research is needed to understand the implications of PMTCT policy and changing protocols, particularly option B+. As more pregnant women are encouraged to initiate cART or ARV prophylaxis earlier in pregnancy and continue during breastfeeding [1], some of the factors associated with adherence to PMTCT treatment may change or become more pronounced. It was not clear whether pill burden of prophylaxis or cART earlier in pregnancy presents a growing barrier to ARV use during this period, as the two studies investigating this issue found conflicting results. Health workers may struggle to keep up with changing protocols, although health-worker knowledge was rarely assessed and should be investigated further. This review suggests that early adoption of option B+ may help to overcome some barriers to the uptake of ARVs for PMTCT, for example delayed ANC attendance. However other barriers identified, such as stigma, fear of disclosure of HIV status and lack of support (which may be more problematic with the need to store and take ARV treatments more regularly) as well as health-systems issues (e.g. the need for repeated trips to the clinic to collect drugs, and already strained infrastructure and resources), suggest the need for cautious implementation, reflecting recent debates in the literature [92].

Our assessment of changing barriers over time revealed that stigma, alongside fear of disclosure, remains entrenched across sub-Saharan Africa, while long-standing health-systems issues such as staffing and accessibility continue to hamper uptake of PMTCT ARVs. The lack of progress in addressing these fundamental issues over 10 years since the implementation of PMTCT programmes is disappointing, and points to a lack of commitment by international donors to invest in health infrastructure, while giving preference to funding more readily audited inputs such as drugs. However, it was interesting that knowledge/education and some psychological barriers appeared less often in recent studies, which may suggest that educational efforts and counselling messages could be beginning to have an effect, or that familiarity with the programme is growing (although these results should be interpreted with caution given the small sample size, and frequency that factors were potentially *investigated* could not be determined for all studies).

### Strengths and limitations

Inclusion of both qualitative and quantitative research and triangulation of results were strengths of this review. The quality appraisal, a weak area of existing systematic reviews incorporating qualitative literature, allowed us to consider study quality when reporting the strength of evidence, and to assess the overall quality of work in this area. The quality assessment tools provide a methodological contribution. Interpretation is limited by the fact that included studies were almost exclusively observational designs, with possible (residual) confounding resulting in over or underestimation of some of the reported associations. The diversity of factors explored in quantitative studies and range of designs impeded a quantitative synthesis of evidence; instead we aimed to describe the results in detailed narrative and tables, and synthesize findings through thematic analysis. Reasons for heterogeneity in quantitative results may include lack of power (sub-analyses), factors varying in importance in different settings, differences in local PMTCT guidelines, different outcome definitions or explanatory factor categories, or spurious results (small *p*-values) generated by chance when many risk factors were analyzed. Some of the quantitative outcome measurements were based on self-reports by the mother or health worker, suggesting the possibility for recall bias.

Only English language publications were searched and studies that found no evidence for/negative associations may not have been published (publication bias). Selection bias is also possible as one reviewer screened the majority of references, but there was a high level of agreement on the double-screened random sample.

This review is limited in its scope, as it does not cover barriers to the whole PMTCT “cascade” or other programme “prongs.” However, many of the identified barriers are cross-cutting and could have implications for other PMTCT “prongs” or cascade steps, or other HIV/maternal and child health services more broadly.

## Conclusions

This review revealed many factors that contribute to the low uptake of ARVs for PMTCT in sub-Saharan Africa, at the level of individuals, their community and health systems. Fundamental health-systems issues such as staffing and service accessibility, along with community-level factors of stigma, fear of disclosure and lack of partner support, emerged consistently across a range of settings in sub-Saharan Africa, and continue to plague PMTCT programmes over 10 years since their introduction. The potential of PMTCT programmes to virtually eliminate vertical transmission of HIV will remain elusive unless these barriers are tackled, and coverage is extended to (as yet) unreached vulnerable populations; an under-researched area. Solutions must involve local communities given the prominence of community-level factors in this analysis. Health-systems strengthening, enhanced counselling, community/partner support, male involvement and educational strategies also have the potential to improve uptake. Packages of solutions to address barriers at different levels are likely to be the most effective.
